# Robotic Arm Alignment System for Electrode Implantation in Invasive Epilepsy Monitoring: Clinical Experience and Accuracy in a Consecutive Patient Series with Comparison to the Literature

**DOI:** 10.3390/jcm15186948

**Published:** 2026-09-08

**Authors:** Julia Shawarba, Johannes Zielke, Ekaterina Pataraia, Florian A. Mayer, Karl Roessler

**Affiliations:** 1 Neurosurgical Department, Medical University Vienna, 1090 Vienna, Austria; julia.shawarba@meduniwien.ac.at; 2 Comprehensive Center for Clinical Neurosciences and Mental Health, Medical University Vienna, 1090 Vienna, Austria; johannes.zielke@meduniwien.ac.at (J.Z.); ekaterina.pataraia@meduniwien.ac.at (E.P.); florian.a.mayer@meduniwien.ac.at (F.A.M.); 3 Department of Pediatrics and Adolescent Medicine, Medical University Vienna, 1090 Vienna, Austria; 4 Department of Neurology, Medical University Vienna, 1090 Vienna, Austria

**Keywords:** stereoelectroencephalography, robotic surgery, stereotactic accuracy, depth electrodes, epilepsy surgery, Brainlab Cirq

## Abstract

**Objectives:** Robot-assisted stereoelectroencephalography (SEEG) is increasingly used for invasive presurgical evaluation in drug-resistant epilepsy. Feasibility and accuracy data for robotic alignment systems remain limited. We analyzed our initial institutional experience and compared the results with the contemporary literature. **Methods:** We retrospectively analyzed nine consecutive patients who underwent SEEG depth electrode implantation using the Brainlab Cirq robotic alignment system and intraoperative MRI for registration. Demographic and operative variables were extracted from institutional records. Implantation accuracy was measured on fused postoperative imaging as Euclidean entry point error (EPE) and target point error (TPE). A PubMed-focused literature screen was performed to identify studies reporting robotic SEEG implantation accuracy and/or efficiency metrics for comparison. **Results:** Nine patients underwent implantation of 69 SEEG electrodes. Mean age was 21.7 ± 11.4 years. A mean of 7.7 ± 2.1 electrodes was implanted per patient. Mean operative time was 120.2 ± 31.9 min, corresponding to 16.0 ± 3.4 min per electrode. Mean EPE was 1.36 ± 0.60 mm and mean TPE was 1.79 ± 0.69 mm. Moreover, the maximum projected skull-entry angle was not independently associated with either EPE or TPE. In comparison with published robotic SEEG series and meta-analyses, our accuracy results fall within the established high-precision range and compare favorably with the most recent robotic-arm-specific data. **Conclusions:** In this initial consecutive series, our robotic arm alignment system enabled accurate SEEG implantation with sub-2-mm mean target error. These findings support robotic arm systems as effective robotic alignment platforms for invasive epilepsy monitoring, while larger multicenter studies remain necessary to define comparative accuracy, learning curve effects, and complication profiles.

## 1. Introduction

Stereoelectroencephalography (SEEG) has become a central method for invasive presurgical evaluation of patients with drug-resistant focal epilepsy, particularly when noninvasive data are discordant, when the suspected epileptogenic network is deep or multilobar, or when bilateral exploration is required [1,2]. Unlike subdural grid implantation, SEEG permits three-dimensional sampling of cortical and subcortical structures through multiple minimally invasive trajectories.

The clinical value of SEEG depends not only on the anatomo-electroclinical hypothesis, but also on the precision with which planned trajectories are executed. Electrode deviation may reduce sampling accuracy, compromise interpretation of seizure onset and propagation, and increase the theoretical risk of vascular or eloquent-structure injury. Consequently, entry point error, target point error, angular deviation, and depth error have become standard quantitative endpoints in studies of SEEG implantation accuracy [3,4]. Robotic platforms were introduced to improve workflow reproducibility and efficiency in stereotactic depth electrode implantation. Established systems such as ROSA and Neuromate have shown high accuracy in large clinical series and systematic reviews [5,6,7,8]. More recently, compact robotic alignment systems have been incorporated into neuronavigation-based workflows. The investigated Brainlab Cirq system is a table-mounted robotic alignment arm designed to guide instruments along preplanned trajectories while maintaining the ergonomics of a navigation-integrated operating room environment [9,10].

However, clinical data on Cirq-guided SEEG implantation remain sparse. The present study therefore analyzes the accuracy and workflow characteristics of the first nine consecutive patients treated at our institution using Cirq-guided SEEG implantation and compares these results with available PubMed-indexed literature.

## 2. Materials and Methods

### 2.1. Study Design and Patient Cohort

This retrospective single-center study included nine consecutive patients who underwent SEEG depth electrode implantation with the Brainlab Cirq robotic alignment system for presurgical evaluation of drug-resistant epilepsy (demographic and operative characteristics are displayed in Table 1). The mean age at surgery was 21.7 ± 11.4 years (median, 17 years; range, 12–45 years). Patients were included if SEEG implantation was performed with Cirq combined with intraoperative MRI (Siemens Skyra, Erlangen, Germany) and the NORAS head coil, which served as both a head-fixation device and an MRI receiver coil (LUCY, NORAS, Würzburg, Germany). Pre- and postoperative MRI was available for accuracy assessment in every patient. The cohort was analyzed at the patient level for demographic and operative parameters and at the electrode level for accuracy endpoints. All patients underwent SEEG following multidisciplinary epilepsy surgery evaluation because noninvasive investigations were insufficient to reliably delineate the seizure onset zone. The cohort represented a heterogeneous spectrum of focal epilepsies, including suspected temporal, temporo-insular, frontal, and more broadly hemispheric or structurally associated epileptogenic networks. In several patients, conventional MRI did not reveal a definite epileptogenic lesion, and the indication and electrode implantation strategy were therefore based on the integration of seizure semiology, video-EEG findings, functional imaging, and other available presurgical investigations. Other patients had structural abnormalities or a history of previous neurosurgical treatment, including prior temporal lobe surgery or resection of an intracranial tumor. In patients with previous epilepsy surgery, recurrent or persistent seizures prompted further invasive evaluation to delineate the residual or alternative epileptogenic network. The individual SEEG implantation strategy was determined according to the presumed epileptogenic network following interdisciplinary case discussion. A total of 69 depth electrodes were implanted, with a mean of 7.7 ± 2.1 electrodes per patient (range, 6–12).

### 2.2. Surgical Planning and Implantation Workflow

All trajectories were planned preoperatively at the Brainlab workstation in stereotaxy mode (Brainlab, Munich, Germany) according to the individual anatomo-electroclinical hypothesis. Planning aimed to sample the suspected seizure onset zone and propagation network while avoiding vascular structures and eloquent regions. For intraoperative imaging and navigation registration, the patient’s head was immobilized in the NORAS head coil, which combines rigid head fixation with a dedicated MRI receiver coil. The head coil is equipped with optical reference markers compatible with the Brainlab navigation system. Following acquisition of the intraoperative planning MRI with the patient’s head immobilized in the coil, the imaging dataset could be directly registered to the navigation system using these predefined reference markers. This marker-based registration provided a rigid and reproducible spatial reference between the patient, the intraoperative MRI dataset, and the navigation system, thereby avoiding reliance on conventional skin-surface registration and its associated potential for registration error [9,10,11]. Following registration, the Cirq robotic alignment arm was integrated with the Brainlab navigation system and used to align the instrument guide with each planned trajectory. After robotic alignment, burr holes and anchoring bolts were placed according to the institutional SEEG workflow, followed by insertion of the depth electrodes to the predefined target depth (Dixi Medical, Marchaux, France).

### 2.3. Implantation Technique

All procedures were performed under general anesthesia using the NORAS head-coil-based image-guided stereotactic workflow. Electrode trajectories were planned individually according to the anatomo-electroclinical hypothesis established during multidisciplinary epilepsy surgery evaluation. Depending on the presumed epileptogenic network, trajectories sampled temporal and mesial temporal structures, frontal regions, insular and opercular cortex, parietal regions, cingulate cortex, or combinations of these areas. In patients with previous resections or structural lesions, trajectories were additionally designed to sample the margins of the resection cavity or lesion and the surrounding potentially epileptogenic network.

For intraoperative imaging and navigation registration, the patient’s head was rigidly immobilized in the NORAS head coil, which also served as the MRI receiver coil and was equipped with optical reference markers compatible with the Brainlab navigation system. Following acquisition of an intraoperative contrast-enhanced planning MRI with the head maintained in the operative position, the image dataset was registered to the navigation system using the predefined reference geometry of the head coil. This provided a rigid spatial relationship between the patient’s head, the imaging dataset, and the navigation system and avoided reliance on conventional skin-surface registration. The preoperatively planned trajectories were fused with the intraoperative MRI dataset and reviewed before implantation. Particular attention was paid to cortical and intracranial vascular structures, and trajectories were modified when necessary to avoid vascular conflicts. The Brainlab Cirq robotic alignment arm (Brainlab, Munich, Germany) was subsequently registered to the navigation system and brought into the operative field. For each electrode, the corresponding trajectory was selected in the navigation software, the robotic arm was brought into approximate position, and robotic fine alignment was performed to orient the instrument guide along the planned trajectory.

Following sterile preparation, a small stab incision was made at the planned entry point and the drilling guide was advanced to the skull surface. A twist-drill craniostomy was created under irrigation, followed by placement of an anchoring bolt. The dura was coagulated and opened through the bolt trajectory. The distance from the outer edge of the bolt to the predefined intracranial target was measured using the navigation plan. A rigid stylet of the corresponding length was then advanced along the trajectory to prepare the implantation tract and subsequently removed. The DIXI Medical depth electrode (DIXI Medical, Chaudefontaine, France) was inserted to the same predefined depth and secured by tightening the fixation cap of the anchoring bolt. This sequence was repeated for each planned trajectory. The number, orientation, and anatomical distribution of electrodes varied according to the individual electroclinical hypothesis. Across the nine procedures, six to twelve electrodes were implanted per patient. Implantations included orthogonal and oblique trajectories targeting frontal, temporal and mesial temporal, insular/opercular, parietal, and cingulate structures. Some trajectories traversed or terminated in deep targets such as the amygdala, hippocampus, or insula, whereas others were directed toward cortical or juxtacortical targets. In patients with previous surgery or structural lesions, electrodes were positioned along resection margins or lesion boundaries to investigate the surrounding epileptogenic network.

After completion of electrode placement, an intraoperative MRI was obtained with the electrodes in situ to verify electrode position and screen for procedure-related intracranial complications. The intraoperative MRI dataset was subsequently used for quantitative assessment of implantation accuracy by comparing the planned and actual electrode trajectories.

### 2.4. Accuracy Measurement

Postoperative imaging was fused with the preoperative planning dataset. For each electrode, the planned entry and target coordinates were compared with the corresponding postoperative electrode trajectory. Entry point error was defined as the distance between the planned and actual cortical/skull entry point. Target point error was defined as the distance between the planned target coordinate and the postoperative electrode tip. Errors are reported in millimeters, consistent with commonly used SEEG accuracy endpoints [1,4,12]. Additionally, EPE and TPE were measured in the fused volumetric imaging dataset and therefore represent three-dimensional Euclidean displacement magnitudes. Formally, the entry displacement vector is ΔE = (ΔX_E, ΔY_E, ΔZ_E), with EPE = √(ΔX_E^2^ + ΔY_E^2^ + ΔZ_E^2^), and the target displacement vector is ΔT = (ΔX_T, ΔY_T, ΔZ_T), with TPE = √(ΔX_T^2^ + ΔY_T^2^ + ΔZ_T^2^).

### 2.5. Literature Screen

A PubMed-focused literature screen was performed using combinations of the terms stereoelectroencephalography, SEEG, robot-assisted, robotic, ROSA, Neuromate, Cirq, accuracy, entry point error, target point error, radial error, and depth error. Studies were prioritized if they reported quantitative implantation accuracy, operative efficiency, complications, or direct comparisons between robotic and nonrobotic techniques. Systematic reviews and meta-analyses were included to contextualize pooled estimates and the reported between-study heterogeneity [1,3,5,6,7].

### 2.6. Statistical Analysis

Continuous variables are reported as mean ± standard deviation, median, and range where appropriate. Electrode-level accuracy metrics are reported across all implanted electrodes. Pearson correlation was used descriptively to explore the relationship between EPE and TPE. No formal hypothesis testing against literature-derived cohorts was performed because source studies were heterogeneous in registration workflow, imaging modality, fixation method, accuracy definitions, and reporting format, a limitation repeatedly emphasized in systematic reviews of SEEG accuracy [1,3,5,6,7].

## 3. Results

### 3.1. Patient and Operative Characteristics

Nine consecutive patients were included. Mean patient age was 21.7 ± 11.4 years, with a range of 12 to 45 years. The explored regions included temporal, frontal, insular, parietal, and multilobar networks, reflecting a broad range of invasive epilepsy hypotheses. A total of 69 electrodes were implanted, corresponding to a mean of 7.7 ± 2.1 electrodes per patient. Mean operative time was 120.2 ± 31.9 min, and the mean operative time per electrode was 16.0 ± 3.4 min.

### 3.2. Electrode Implantation Accuracy

**Figure 1:**
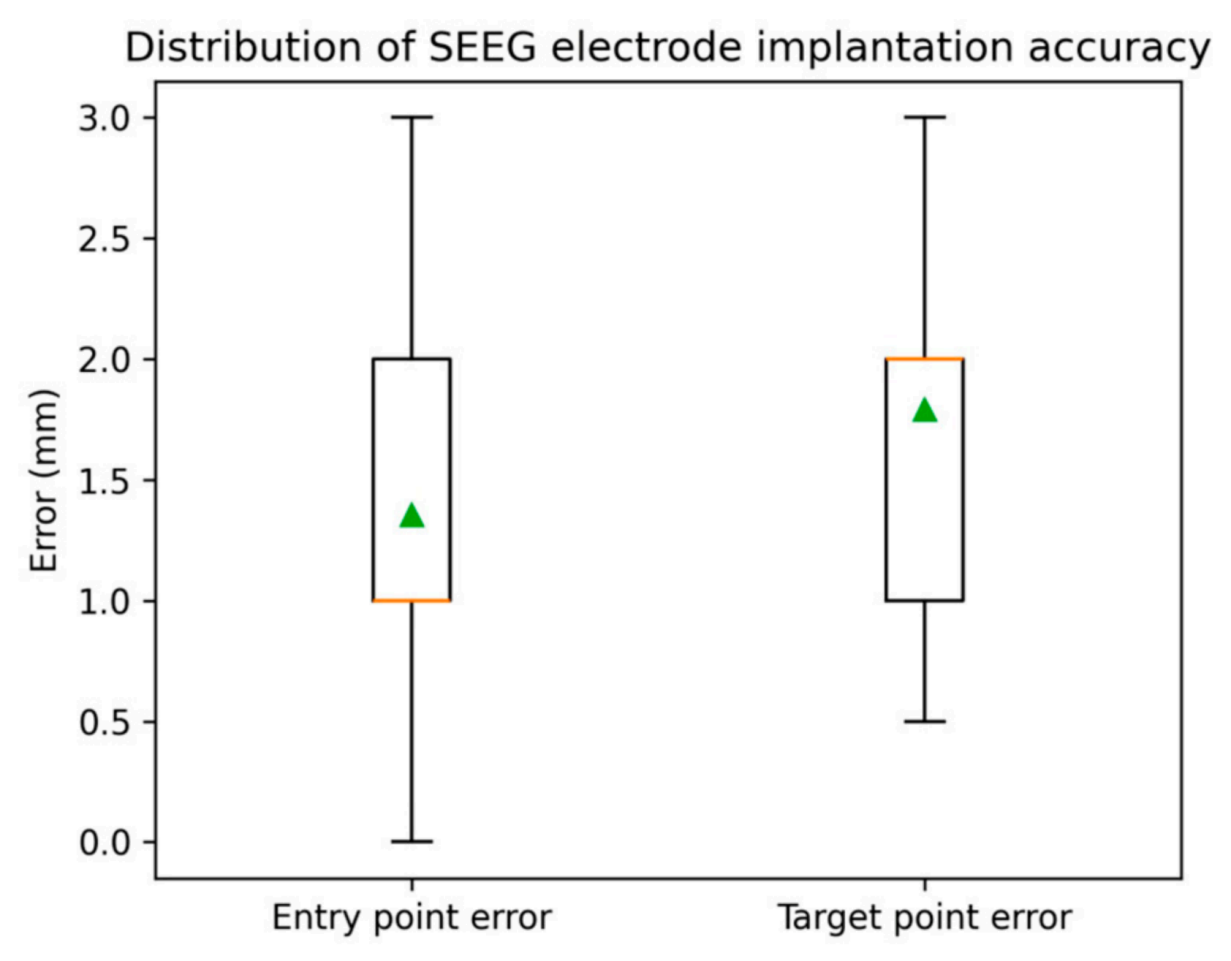
Distribution of electrode implantation errors. Box-and-whisker plots showing entry point error (EPE) and target point error (TPE) across all 69 implanted SEEG electrodes. Boxes represent the interquartile range, horizontal lines indicate the median, and whiskers illustrate the distribution of the observed values. Errors are expressed in millimeters.

**Figure 2:**
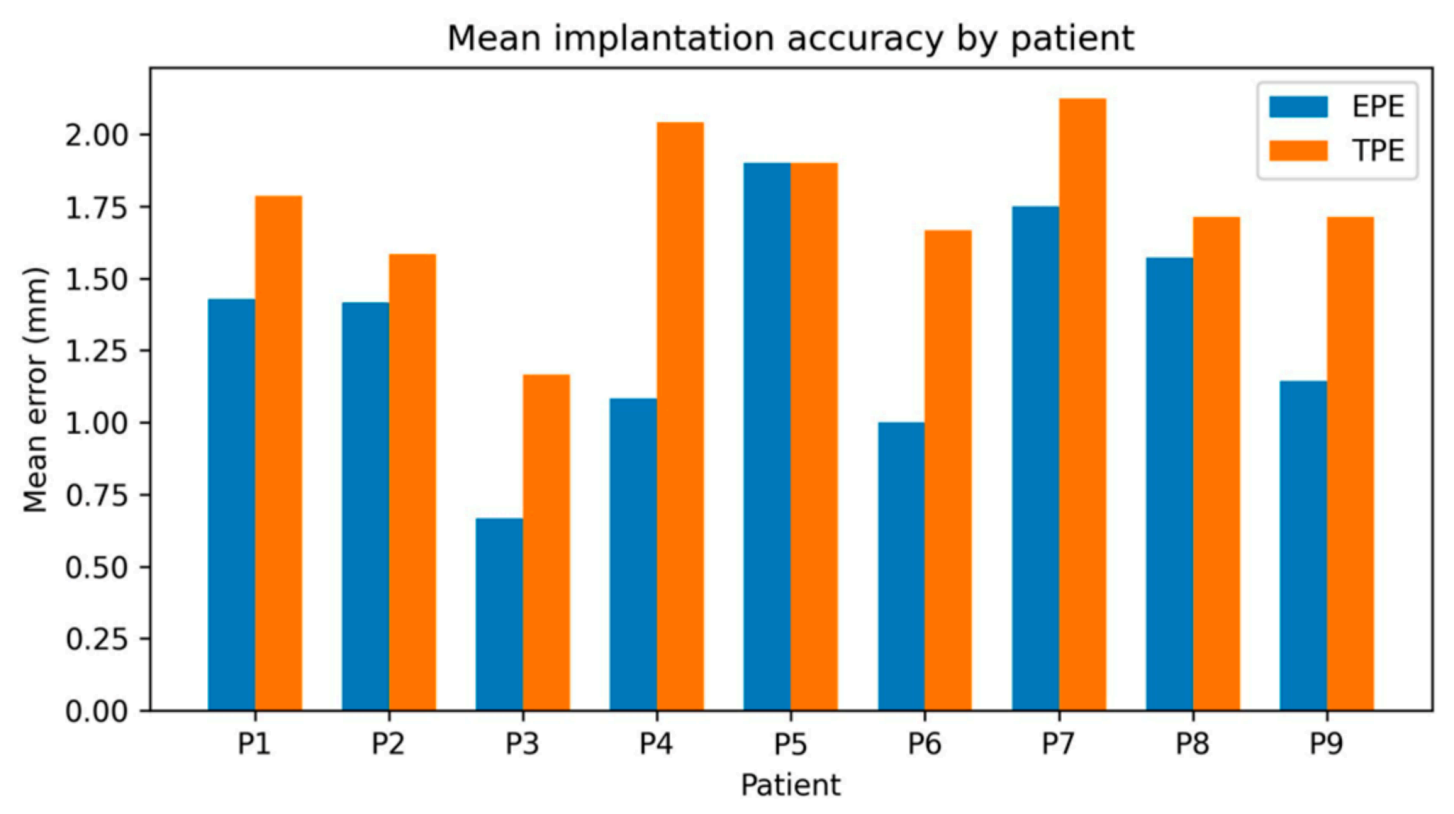
Patient-level implantation accuracy. Mean entry point error (EPE) and target point error (TPE) for each of the nine patients. Bars represent the mean error across all electrodes implanted in the respective patient. The number of implanted electrodes ranged from six to twelve per patient.

**Figure 3:**
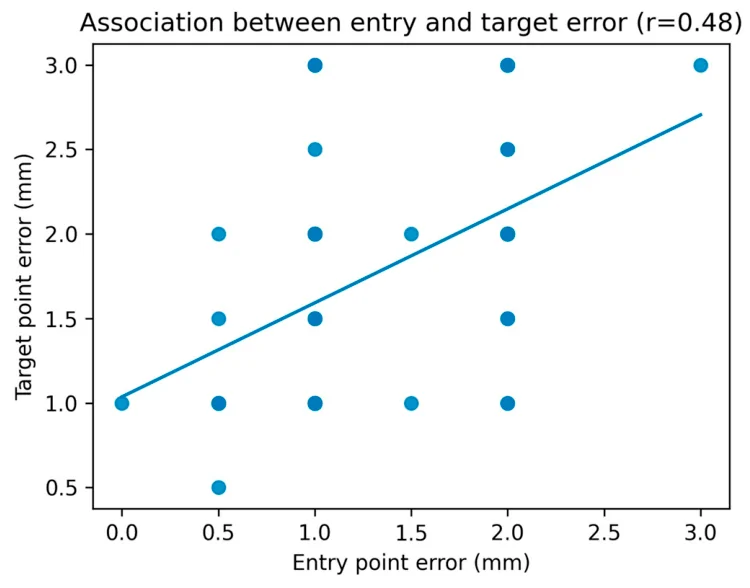
Association between entry point error and target point error. Scatter plot showing electrode-level entry point error (EPE) and target point error (TPE) across all 69 implanted SEEG electrodes. Each point represents one electrode. EPE and TPE demonstrated a moderate positive correlation (Pearson r = 0.48).

Across all 69 implanted electrodes, mean EPE was 1.36 ± 0.60 mm. The median EPE was 1.0 mm, and the observed range was 0.0 to 3.0 mm. Mean TPE was 1.79 ± 0.69 mm, with a median of 2.0 mm and a range of 0.5 to 3.0 mm. The 95% confidence intervals were 1.21–1.50 mm for EPE and 1.63–1.95 mm for TPE (Table 1 and Table 2, Figure 1 and Figure 2). Entry and target error showed a moderate positive correlation (r = 0.48), indicating that larger entry deviations tended to be associated with larger target deviations, Figure 3. In unadjusted electrode-level analysis, inline-2 projected entry angle showed a weak positive correlation with EPE (Pearson r = 0.30, *p* = 0.011; Spearman ρ = 0.29, *p* = 0.014). However, this association was no longer present after accounting for patient-level clustering and between-patient differences (β = 0.014 mm/degree; 95% CI, −0.021 to 0.049; *p* = 0.379). The maximum projected skull-entry angle was not independently associated with EPE (adjusted β = 0.005 mm/degree; 95% CI, −0.023 to 0.034; *p* = 0.679) or TPE (adjusted β = −0.007 mm/degree; 95% CI, −0.030 to 0.017; *p* = 0.537).

### 3.3. Literature Comparison

**Figure 4:**
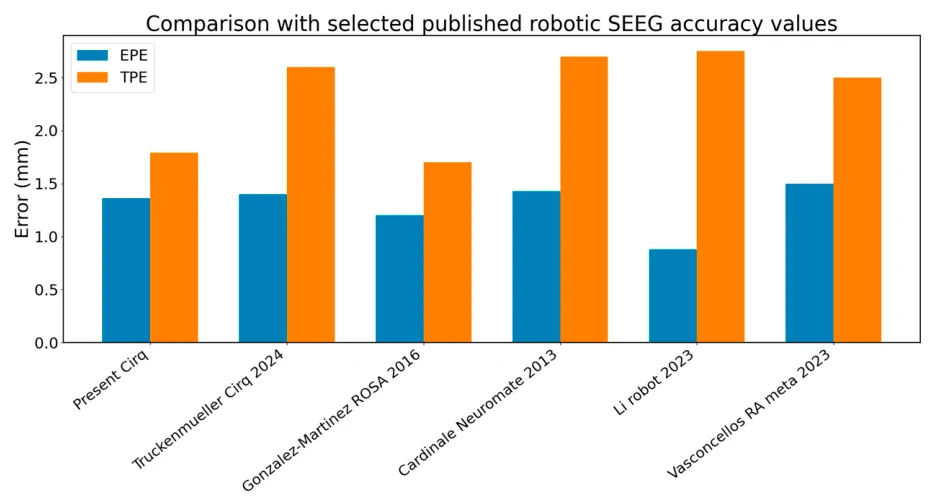
Comparison with published robotic SEEG accuracy data. Mean entry point error (EPE) and target point error (TPE) in the present Brainlab Cirq cohort compared with selected published robotic SEEG series [3,8,10,19,20]. The 1.4-mm EPE and 2.6-mm TPE Cirq values are from Truckenmueller et al. [10]. Cross-study comparisons should be interpreted cautiously because robotic platforms, registration and fixation techniques, imaging modalities, and definitions of implantation accuracy differ across reports [1,3,5,6,7].

The literature screen identified several major topics relevant to the interpretation of our data, Figure 4. First, systematic reviews report robotic SEEG accuracy in the low-millimeter range but also document substantial heterogeneity related to imaging, registration, fixation, and measurement methodology [1,3,5,6,7]. Second, comparative studies generally suggest shorter implantation times with robotic workflows and accuracy that is improved or at least maintained relative to frameless or frame-based approaches [6,7,13,14,15]. Recent pediatric, novel-robot, and frame-based series further highlight safety, accuracy, and workflow determinants across differing populations and stereotactic approaches [16,17,18]. Third, Cirq-specific SEEG evidence remains limited. Truckenmueller et al. reported mean EPE of 1.4 ± 1.2 mm and mean TPE of 2.6 ± 1.6 mm in 31 electrodes implanted during five Cirq-assisted SEEG procedures using automated cone-beam CT registration [10]. Kanaya et al. reported a separate eight-case, 37-electrode Cirq series using surface matching, but their coordinate-based two- and three-dimensional metrics are not directly interchangeable with the EPE and TPE definitions used here [9]. Against this background, the present EPE of 1.36 mm is essentially identical to the value reported by Truckenmueller et al., while the observed TPE of 1.79 mm is lower; these descriptive cross-study differences should not be interpreted as a formal superiority comparison.

## 4. Discussion

The present study demonstrates that SEEG implantation using the Brainlab Cirq robotic alignment system can be performed with high stereotactic accuracy. In 69 electrodes implanted in nine consecutive patients, mean EPE was 1.36 mm and mean TPE was 1.79 mm. These values fall within the low-millimeter accuracy range reported in systematic reviews and large robotic SEEG series [1,3,5,8,19]. Because accuracy definitions and imaging workflows vary across studies, this comparison is contextual rather than confirmatory [1,3,5,6,7].

The most directly comparable Cirq-specific values are those of Truckenmueller et al., who reported mean EPE of 1.4 mm and mean TPE of 2.6 mm [10]. Our EPE is nearly identical, whereas our TPE is lower. Kanaya et al. also documented Cirq-guided SEEG accuracy, but used different coordinate-based two- and three-dimensional measures, which precludes a direct numerical comparison with our EPE and TPE [9]. Differences between reports may reflect cohort composition, trajectory length, target anatomy, imaging fusion, registration workflow, fixation, and measurement method [9,10,11]. The agreement in EPE with Truckenmueller et al. is therefore reassuring, but should be interpreted descriptively rather than as evidence of equivalence or superiority.

Compared with established robotic systems, the present accuracy is also within the ranges reported for ROSA and Neuromate. Large series describe median target errors of approximately 1.7–1.8 mm, while systematic reviews and other cohorts report low-millimeter entry and target deviations with substantial methodological variation [3,5,8,14,19]. Meta-analytic data confirm high overall precision but emphasize that heterogeneous definitions and workflows limit direct ranking of systems [1,3,5,6,7]. Accordingly, the present mean TPE below 2 mm supports the technical feasibility of the Cirq workflow but does not establish superiority over another platform.

Additionally, skull-entry obliquity has been proposed as a potential determinant of stereotactic accuracy because an oblique trajectory may increase the risk of drill skiving or trajectory deflection at the cranial surface; drill slippage at steep skull angles has also been discussed in Cirq-specific experience [9]. In the present cohort, the inline-2 projected entry angle showed a weak positive association with EPE in the unadjusted electrode-level analysis. However, this association disappeared after accounting for the clustering of electrodes within patients, suggesting that the unadjusted finding was predominantly attributable to between-patient differences rather than to a consistent effect of increasing obliquity within individual patients. Moreover, the maximum projected skull-entry angle was not independently associated with either EPE or TPE. These findings suggest that, within the range of entry orientations represented in this series, the Cirq-guided drilling and bolt-based implantation workflow maintained comparable accuracy for more perpendicular and more oblique trajectories. Nevertheless, the absence of a statistically significant association should not be interpreted as evidence of equivalence or as proof that skull-entry angle has no effect. The analysis was limited by the small number of patients and the clustering of multiple electrodes within patients.

Workflow efficiency is another relevant finding. Mean operative time per electrode was 16.0 min. This is comparable with reported robotic workflows and supports the practical utility of a compact table-mounted alignment system. Unlike larger floor-mounted robots, Cirq may be attractive for centers already using Brainlab navigation because it can be incorporated into an existing navigation infrastructure without major changes to the operating room footprint [10]. The moderate correlation between EPE and TPE in our cohort is consistent with the concept that small proximal deviations may propagate along long intracranial trajectories. Lu et al. similarly emphasized the contribution of entry error, depth error, and angular deviation to target accuracy [4]. This reinforces the importance of accurate registration, rigid head fixation, careful robotic alignment, and stable instrument handling throughout drilling, bolt placement, and electrode insertion.

### 4.1. Complications

No clinically significant hemorrhages, infections, neurological deficits, electrode revisions, or technical failures occurred in the study cohort. Although this finding is reassuring, systematic reviews and large series show that hemorrhage and other complications are uncommon but not absent; a nine-patient cohort is therefore insufficient to establish comparative safety [1,3,5,8,19].

### 4.2. Limitations

This study has important limitations. First, the relatively small sample size limits the statistical power of the analysis and the precision of the reported estimates. In particular, uncommon technical or procedural deviations may not be adequately represented, and the ability to identify factors associated with implantation accuracy is limited. Therefore, the observed accuracy metrics should be interpreted as descriptive findings and require confirmation in larger cohorts. Second, the retrospective, single-center design may introduce selection and information bias and may limit the generalizability of our findings. All procedures were performed within a single institutional environment, with center-specific surgical workflows, imaging protocols, registration procedures, and operator experience. Consequently, the results may not fully reflect the performance achievable across centers with different levels of experience, patient populations, or technical workflows. Prospective multicenter studies with standardized imaging and accuracy assessment protocols would therefore be valuable to confirm the reproducibility and external validity of these findings. In addition, no direct institutional control group was available, precluding a direct within-center comparison of implantation techniques. Finally, cross-study comparisons are inherently limited by heterogeneous definitions of accuracy, different imaging modalities, variable registration workflows, and inconsistent reporting of mean versus median error, as emphasized in prior systematic reviews and studies of registration methodology [1,3,5,6,7,11].

## 5. Conclusions

In this initial consecutive series, Brainlab Cirq-guided SEEG implantation achieved a mean entry point error of 1.36 mm and a mean target point error of 1.79 mm across 69 electrodes, with a mean implantation time of 16.0 min per electrode. The maximum projected skull-entry angle was not independently associated with either EPE or TPE. The accuracy values lie within the ranges reported for established robotic platforms and are consistent with the limited Cirq-specific literature [5,8,9,10,19]. These findings support the technical feasibility and accuracy of this navigation-integrated robotic alignment workflow, while the small retrospective cohort does not permit claims of comparative superiority or safety. Larger prospective multicenter studies are needed to validate accuracy, complication rates, operative efficiency, and learning-curve effects.

## Figures and Tables

**Table 1:** Demographic and operative characteristics of the nine patients undergoing Cirq-assisted SEEG electrode implantation. Continuous variables are presented as mean ± standard deviation and range, where applicable.

Variable	Value
Patients, n	9
Age, years	21.7 ± 11.4; median 17; range 12–45
Implanted electrodes, n	69
Electrodes per patient	7.7 ± 2.1; range 6–12
Operative time, min	120.2 ± 31.9; range 86–192
Operative time per electrode, min	16.0 ± 3.4; range 10.8–20.8

**Table 2:** Electrode-level implantation accuracy across all 69 SEEG electrodes. Entry point error (EPE) and target point error (TPE) are reported as mean ± standard deviation, median, range, and 95% confidence intervals.

Accuracy Metric	Mean ± SD, mm	Median, mm	IQR, mm	Range, mm	95% CI, mm
Entry point error	1.36 ± 0.60	1.0	1.0–2.0	0.0–3.0	1.21–1.50
Target point error	1.79 ± 0.69	2.0	1.0–2.0	0.5–3.0	1.63–1.95

## Data Availability

The data are available from the corresponding author upon reasonable request.

## Abbreviations

The following abbreviations are used in this manuscript:

SEEG	stereoelectroencephalography
EPE	entry point error
TPE	target point error
MRI	magnetic resonance imaging

## References

[1] Vakharia V.N., Sparks R., O’Keeffe A.G., Rodionov R., Miserocchi A., McEvoy A., Ourselin S., Duncan J. (2017). Accuracy of intracranial electrode placement for stereoelectroencephalography: A systematic review and meta-analysis. Epilepsia.

[2] Iida K., Otsubo H. (2017). Stereoelectroencephalography: Indication and Efficacy. Neurol. Med. Chir..

[3] Vasconcellos F.N., Almeida T., Müller Fiedler A., Fountain H., Santos Piedade G., Monaco B.A., Jagid J., Cordeiro J.G. (2023). Robotic-Assisted Stereoelectroencephalography: A Systematic Review and Meta-Analysis of Safety, Outcomes, and Precision in Refractory Epilepsy Patients. Cureus.

[4] Lu C., Chen S., An Y., Meng F., Wang Y., Wei P., Fan X., Shan Y., Zhao G. (2021). How can the accuracy of SEEG be increased?—An analysis of the accuracy of multilobe-spanning SEEG electrodes based on a frameless stereotactic robot-assisted system. Ann. Palliat. Med..

[5] Kaewborisutsakul A., Chernov M., Yokosako S., Kubota Y. (2024). Usefulness of Robotic Stereotactic Assistance (ROSA^®^) Device for Stereoelectroencephalography Electrode Implantation: A Systematic Review and Meta-analysis. Neurol. Med. Chir..

[6] Abbas A., Sabet H., El Refaei K., AbuHamdia A., Elboraay T., Negida Y., Aldehri M., Alnaami I., Raslan A.M. (2026). Robot-assisted versus frame-based stereoelectroencephalography (sEEG) electrode implantation in drug-resistant epilepsy: A meta-analysis of accuracy, efficiency, and safety. Acta Neurochir..

[7] Gomes F.C., Larcipretti A.L.L., Nager G., Dagostin C.S., Udoma-Udofa O.C., Pontes J.P.M., de Oliveira J.S., de Souza J.H.C., Bannach M.A. (2023). Robot-assisted vs. manually guided stereoelectroencephalography for refractory epilepsy: A systematic review and meta-analysis. Neurosurg. Rev..

[8] Cardinale F., Cossu M., Castana L., Casaceli G., Schiariti M.P., Miserocchi A., Fuschillo D., Moscato A., Caborni C., Arnulfo G. (2013). Stereoelectroencephalography: Surgical methodology, safety, and stereotactic application accuracy in 500 procedures. Neurosurgery.

[9] Kanaya K., Nakamura A., Abe D., Sato Y., Wakabayashi M., Shigehara T., Watanabe D., Yoshizawa Y., Fukuyama T., Horiuchi T. (2025). Clinical experiences and accuracy of stereoelectroencephalography using the robotic arm Cirq. Acta Neurochir..

[10] Truckenmueller P., Früh A., Kissner J.F., Moser N.K., Misch M., Faust K., Onken J., Vajkoczy P., Xu R. (2024). Integration of a lightweight and table-mounted robotic alignment tool with automated patient-to-image registration using robotic cone-beam CT for intracranial biopsies and stereotactic electroencephalography. Neurosurg. Focus.

[11] Spyrantis A., Cattani A., Woebbecke T., Konczalla J., Strzelczyk A., Rosenow F., Wagner M., Seifert V., Kudernatsch M., Freiman T.M. (2019). Electrode placement accuracy in robot-assisted epilepsy surgery: A comparison of different referencing techniques including frame-based CT versus facial laser scan based on CT or MRI. Epilepsy Behav..

[12] Kogias E., Altenmüller D.M., Karakolios K., Egger K., Coenen V.A., Schulze-Bonhage A., Reinacher P.C. (2022). Electrode placement for SEEG: Combining stereotactic technique with latest generation planning software for intraoperative visualization and postoperative evaluation of accuracy and accuracy predictors. Clin. Neurol. Neurosurg..

[13] Sharma J.D., Seunarine K.K., Tahir M.Z., Tisdall M.M. (2019). Accuracy of robot-assisted versus optical frameless navigated stereoelectroencephalography electrode placement in children. J. Neurosurg. Pediatr..

[14] Kojima Y., Uda T., Kawashima T., Koh S., Hattori M., Mito Y., Kunihiro N., Ikeda S., Umaba R., Goto T. (2022). Primary Experiences with Robot-assisted Navigation-based Frameless Stereo-electroencephalography: Higher Accuracy than Neuronavigation-guided Manual Adjustment. Neurol. Med. Chir..

[15] Yao Y., Hu W., Zhang C., Wang X., Zheng Z., Sang L., Shao X., Zhang K. (2023). A comparison between robot-guided and stereotactic frame-based stereoelectroencephalography (SEEG) electrode implantation for drug-resistant epilepsy. J. Robot. Surg..

[16] Lu R., Wang M., Zhang Y., Li H., Zhou Y., Wang Y., Zhao R. (2024). Safety, Accuracy, and Efficacy of Robot-Assisted Stereo Electroencephalography in Children of Different Ages. Neurosurgery.

[17] Kim J., Hong S.W., Dao P.D., Rim J., Yu J.W., Ko A., Kang H.C., Heo K., Kim H.D., Chang J.H. (2025). Safety and efficacy of stereoelectroencephalography using a novel robot based on the center-of-arc principle in patients with medically refractory epilepsy. J. Neurosurg..

[18] Kirchleitner S.V., Quach S., Niedermeyer S., Nerlinger P., Nack A., Vollmar C., Remi J., Zimmermann H., Thon N., Schmutzer-Sondergeld M. (2026). Determinants of accuracy in frame-based stereoelectroencephalography: The role of experience, workflow, and selective use of guiding devices. Front. Neurol..

[19] González-Martínez J., Bulacio J., Thompson S., Gale J., Smithason S., Najm I., Bingaman W. (2016). Technique, Results, and Complications Related to Robot-Assisted Stereoelectroencephalography. Neurosurgery.

[20] Li P., Zhou Y., Zhang Q., Yang Y., Wang M., Zhu R., Li H., Gu S., Zhao R. (2023). Frameless robot-assisted stereoelectroencephalography-guided radiofrequency: Methodology, results, complications and stereotactic application accuracy in pediatric hypothalamic hamartomas. Front. Neurol..

